# Deploying and Optimizing Embodied Simulations of Large-Scale Spiking Neural Networks on HPC Infrastructure

**DOI:** 10.3389/fninf.2022.884180

**Published:** 2022-05-19

**Authors:** Benedikt Feldotto, Jochen Martin Eppler, Cristian Jimenez-Romero, Christopher Bignamini, Carlos Enrique Gutierrez, Ugo Albanese, Eloy Retamino, Viktor Vorobev, Vahid Zolfaghari, Alex Upton, Zhe Sun, Hiroshi Yamaura, Morteza Heidarinejad, Wouter Klijn, Abigail Morrison, Felipe Cruz, Colin McMurtrie, Alois C. Knoll, Jun Igarashi, Tadashi Yamazaki, Kenji Doya, Fabrice O. Morin

**Affiliations:** ^1^Robotics, Artificial Intelligence and Real-Time Systems, Faculty of Informatics, Technical University of Munich, Munich, Germany; ^2^Simulation and Data Lab Neuroscience, Jülich Supercomputing Centre (JSC), Institute for Advanced Simulation, JARA, Forschungszentrum Jülich GmbH, Jülich, Germany; ^3^Swiss National Supercomputing Centre (CSCS), ETH Zurich, Lugano, Switzerland; ^4^Neural Computation Unit, Okinawa Institute of Science and Technology Graduate University, Okinawa, Japan; ^5^Department of Excellence in Robotics and AI, The BioRobotics Institute, Scuola Superiore Sant'Anna, Pontedera, Italy; ^6^Department of Computer Architecture and Technology, Research Centre for Information and Communication Technologies, University of Granada, Granada, Spain; ^7^Image Processing Research Team, Center for Advanced Photonics, RIKEN, Wako, Japan; ^8^Computational Engineering Applications Unit, Head Office for Information Systems and Cybersecurity, RIKEN, Wako, Japan; ^9^Graduate School of Informatics and Engineering, The University of Electro-Communications, Tokyo, Japan; ^10^Jülich Research Centre, Institute of Neuroscience and Medicine (INM-6), Institute for Advanced Simulation (IAS-6), JARA BRAIN Institute I, Jülich, Germany; ^11^Computer Science 3-Software Engineering, RWTH Aachen University, Aachen, Germany; ^12^Center for Computational Science, RIKEN, Kobe, Japan

**Keywords:** spiking neural networks, embodiment, Neurorobotics Platform, high performance computing (HPC), NEST, musculoskeletal modeling, large-scale brain simulation, parallel computing

## Abstract

Simulating the brain-body-environment trinity in closed loop is an attractive proposal to investigate how perception, motor activity and interactions with the environment shape brain activity, and vice versa. The relevance of this embodied approach, however, hinges entirely on the modeled complexity of the various simulated phenomena. In this article, we introduce a software framework that is capable of simulating large-scale, biologically realistic networks of spiking neurons embodied in a biomechanically accurate musculoskeletal system that interacts with a physically realistic virtual environment. We deploy this framework on the high performance computing resources of the EBRAINS research infrastructure and we investigate the scaling performance by distributing computation across an increasing number of interconnected compute nodes. Our architecture is based on requested compute nodes as well as persistent virtual machines; this provides a high-performance simulation environment that is accessible to multi-domain users without expert knowledge, with a view to enable users to instantiate and control simulations at custom scale via a web-based graphical user interface. Our simulation environment, entirely open source, is based on the Neurorobotics Platform developed in the context of the Human Brain Project, and the NEST simulator. We characterize the capabilities of our parallelized architecture for large-scale embodied brain simulations through two benchmark experiments, by investigating the effects of scaling compute resources on performance defined in terms of experiment runtime, brain instantiation and simulation time. The first benchmark is based on a large-scale balanced network, while the second one is a multi-region embodied brain simulation consisting of more than a million neurons and a billion synapses. Both benchmarks clearly show how scaling compute resources improves the aforementioned performance metrics in a near-linear fashion. The second benchmark in particular is indicative of both the potential and limitations of a highly distributed simulation in terms of a trade-off between computation speed and resource cost. Our simulation architecture is being prepared to be accessible for everyone as an EBRAINS service, thereby offering a community-wide tool with a unique workflow that should provide momentum to the investigation of closed-loop embodiment within the computational neuroscience community.

## 1. Introduction

While theories exist that describe how brain architecture and neuronal activity support human-specific, higher-level cognitive abilities such as common sense, capacity for generalization and self-awareness, their experimental validation *in vivo* is usually impossible for both technical (e.g., lack of reproducibility, observability and perturbability) and ethical reasons. As such, simulating the human brain becomes necessary in order to test data-driven hypotheses coming from theoretical neuroscience regarding the structure-function-activity trifecta, and thus establish the link between these in an ethical, reproducible and fully observable manner.

In particular, it is only through simulation that the functional capacity of a given brain model can be consistently evaluated at multiple scales and under various operating conditions, or that the individual contribution of its sub-components to the emergence of advanced cognitive functions can be teased apart. In short, as Nobel physicist Richard Feynman concluded, “what I cannot create I do not understand.” Not just any isolated simulation will do, though. To have any relevance to data collected from living beings, the simulated brain must be afforded with the possibility to interact with a dynamic, physically realistic and sensory-rich environment. This is what we refer to as embodiment. Only then can the simulated neuronal activity be expected to somewhat match, even to a limited extent, that of an actual brain in natural settings. This aspect is therefore essential when studying cognitive mechanisms that involve sensorimotor integration or motor control.

Such an embodied simulation framework must be able to simulate the brain at scale in order to capture the contributions of multiple brain regions involved in goal-directed actions, and to account for the effects of various learning mechanisms, from single synapses up to network effects of different neural populations. It requires significant computing capabilities and a distributed architecture to cope with the highly parallel, resource-intensive nature of large-scale neuronal network simulations, as well as features that allow interactive experimentation while keeping brain and body simulation in sync.

We demonstrate a prototype for a simulation service on the EBRAINS research infrastructure; this prototype enables users to run custom embodied large-scale brain simulations through the Neurorobotics Platform (NRP), the component of EBRAINS dedicated to closed-loop neuroscience. Implemented with the NEST simulator for large-scale spiking neural networks, these brain simulations are run in a distributed manner on a variable allocation of high-performance computing (HPC) nodes of the supercomputer Piz Daint. Within this framework, large-scale biologically plausible neuronal networks with multiple regions are simulated in NEST and interconnected with a physics simulation of a musculoskeletal system in Gazebo. A dedicated graphical user interface in the NRP frontend enables anyone entitled to adequate compute resources on EBRAINS to schedule jobs on the Piz Daint supercomputer at the Swiss National Supercomputing Center (CSCS) and to launch new NRP instances. This process enables users to run, control and interact with embodied simulation experiments online as intuitively as is possible using local installations of the NRP, but backed by the considerable computing power of Piz Daint.

## 2. State of the Art

### 2.1. Large-Scale Neuronal Simulations on HPC Infrastructure

Several tools for the simulation of spiking neurons and networks thereof have been developed. They allow to model a high degree of biological plausibility but differ in their focus on different aspects of the biological models or the technology they use. Highlighting a few, NEURON (Hines and Carnevale, 1997; Awile et al., 2022) GENESIS (Bower and Beeman, 2007) and Arbor (Abi Akar et al., 2019) allow the modeling of complex compartmental neurons and are tailored to the simulation from the sub-cellular level to networks, while the *Open Source Brain* (Gleeson et al., 2019) provides functionalities for visualization and focuses on user collaboration and accessibility of neuron models and networks.

Due to the improved availability of compute resources for neuroscience research through programs like the Human Brain Project's Fenix/ICEI^1^, or the Neuroscience Gateway^2^ and advances in simulation technology (e.g., Jordan et al., 2018; Kumbhar et al., 2019), it became routinely possible for computational neuroscientists to run large-scale simulations of spiking neuronal networks with great efficiency. Most modern neuronal network simulators achieve linear scaling for a large range of simulations of neuroscientific models and have thus opened the way to increased model sizes and more complex learning paradigms.

Meanwhile, a large number of projects are making use of these technological developments, which also resulted in a number of large-scale modeling publications (Markram et al., 2015; Senk et al., 2018; Igarashi et al., 2019; Billeh et al., 2020). Many of the studies are scaling to considerable portions of the world's largest supercomputers and reach far beyond the simple random balanced network that has been the norm in the field for many years. By integrating data from multiple neuroanatomical and electrophysiological sources, they enable the study of biological phenomena with an unprecedented level of detail. At the same time, the developers of the simulation tools are facing new challenges when it comes to coupling simulators amongst each other to increase the realism of the simulated models and to allow for an integration of physics simulators in scenarios such as those described in the present work.

### 2.2. Simulations of Spiking Neural Networks Controlling Virtual Embodied Agents

Previous works involving simulations of spiking neural networks connected to an embodied agent (either a robot or a musculoskeletal system) have mostly aimed at understanding motor control in the brain in relation to sensorimotor integration. Many of them focused on functional performance and were often carried out in a robotic context (e.g., Gilra and Gerstner, 2018; Bahuguna et al., 2019; Angelidis et al., 2021). Others more specifically investigated the robustness, versatility and capacity for adaptation of biological motor systems, for which there is still no satisfactory mechanistic explanatory framework. As an example, DeWolf et al. (2016) used the Neural Engineering Framework (NEF; Eliasmith and Anderson, 2004) to implement a multi-area brain model capable of controlling a three-link arm which also successfully exhibited adaptation to changes in arm dynamics and kinematic structure.

Other research efforts found in the literature involving spiking neural networks controlling a body were about replicating specific features of biological motor systems, with a focus usually placed more on simple movement generation rather than complex, behaviorally-relevant interactions with the environment (e.g., Allegra Mascaro et al., 2020; Fernándes et al., 2021; Kalidindi et al., 2021). In order to achieve task completion, these often involved some network training/optimization process, be it biologically realistic (e.g., STDP in Fernándes et al., 2021) or derived from AI approaches (back-propagation through time in Kalidindi et al., 2021). As for the simulation of musculoskeletal systems, it usually attempted to remain as biologically realistic as possible (e.g., through the use of Hill muscle models), but the experimental implementation did not provide straightforward means for reuse and reproducibility testing. Very few efforts reported in the literature besides the Neurorobotics Platform (see Section 2.3 below) actually focused on this aspect, which makes them all the more remarkable (e.g., Jordan et al., 2019). The latter introduces a toolchain to connect NEST with OpenAIGym making use of the MUSIC interface (Djurfeldt et al., 2010; Brocke, 2020). In Bahuguna et al. (2019), MUSIC is used to connect NEST with Gazebo. The Neurorobotics Platform connects physics and neural simulations directly using Nengo (Angelidis et al., 2021) or NEST (Allegra Mascaro et al., 2020).

The brain-body-environment trinity for different species at different levels of complexity from single body limbs to full body simulations has been simulated in multiple frameworks. The most popular example for invertebrates can be found in the OpenWorm platform (Szigeti et al., 2014; Sarma et al., 2018), which is made for the complete simulation of the Caenorhabditis elegans modeled with both its full body using fluid-simulation dynamics and the full neural network consisting of 302 neurons. A whole body simulation model including environment interaction of a vertebrate is found in Ferrario et al. (2021) with the simulation of a tadpole and serves as an experiment platform for research questions ranging from decision-making to movement generation. While both of the aforementioned simulation platforms are specialized for the given species, AnimatLab (Cofer et al., 2010) is a more generic simulation platform, which allows simulations of a wide range of vertebrates and invertebrates. Cofer et al. (2010) described a human arm flexion as an example.

The most complex work connecting a spiking model of the brain to a body can be found in Yamada et al. (2016). It describes a system encompassing a musculoskeletal model of human fetus at 32 weeks of gestation, a brain (2.6 million leaky integrate-and-fire spiking neurons and 5.3 billion synaptic connections) and some limited environmental modeling, which was used to comparatively study touch-driven cortical learning via limited embodied interactions under intrauterine and extrauterine environmental conditions.

### 2.3. Neurorobotics Platform

The HBP Neurorobotics Platform (NRP) is the backbone of the EBRAINS Closed-Loop Neuroscience service (Knoll et al., 2016). It provides access to a physically realistic simulated environment within which users can simulate and use all kinds of neural models (including spiking neural networks running on neuromorphic chips) composed into functional architectures, and connected to physical incarnations (musculoskeletal models or robotic systems). The simulation of the environment is carried out in Gazebo, an open-source robotic simulator. The neural models can be implemented using one of several frameworks, such as the software simulators NEST (Gewaltig and Diesmann, 2007) or Nengo (Bekolay et al., 2014), or the neuromorphic system SpiNNaker (Furber et al., 2014). The execution of the various simulators involved in a given NRP simulation is orchestrated by a dedicated component referred to as the Closed-Loop Engine (CLE). The connections between the simulated agents' bodies and brains are entirely user-configurable, within the limitations imposed by the application programming interfaces (APIs) of the various simulators. The details of the connections are established through a dedicated framework of so-called Transfer Functions, which are responsible for the conversion and processing of data in transit for seamless recurrent communication between simulators. The NRP can be downloaded and installed locally for maximum experimental convenience, or accessed online in order to leverage the EBRAINS HPC infrastructure for large-scale experiments, as in the present case.

The functional connection of neural models to embodied agents allows neuroscientists to explore how the brain performs a number of tasks in closed loop, from lower-level sensorimotor tasks to higher cognitive functions (e.g., contextual awareness, decision making, etc.). The NRP thus enables cognitive and computational neuroscientists to explore the relationships that exist between the architectural characteristics of neural circuitry (usually constrained by anatomical and connectome data), neuronal dynamics (activity at either population or single-cell level), and their function expressed as the overt behavior of an embodied agent. Furthermore, *in silico* simulation provides a level of control over experimental parameters that enables studies that would be either technically impossible or ethically unacceptable. For example, only in simulation one can fully observe the effect of knocking out a particular ion channel in a specific neuronal sub-population with perfect efficiency, or carry out lesioning studies with perfect reproducibility. As such, the NRP provides a unique enabling platform to probe the functional consequences of e.g., stroke (Allegra Mascaro et al., 2020) or pharmacological tampering on the central nervous system. It is therefore a valuable tool to elucidate outstanding questions around motor control in both health and disease. However, until the work reported in the present paper, the NRP was run either locally or as a cloud service (i.e., on virtual machines). As such, the size of simulations that could be run on the NRP was limited by the typical computing resources of standard computers or virtual machines.

### 2.4. The Neural Simulation Tool NEST

NEST is a simulator for large networks of spiking neurons connected by phenomenological synapse models. It supports hybrid parallel simulations using threading within CPUs and the message passing interface (MPI) across multiple CPUs and computing nodes. In previous studies, NEST has shown excellent scaling over a large number of architectures even on the world's largest supercomputers (Kunkel et al., 2014; Jordan et al., 2018). The details of the parallelization are entirely transparent to the users, who do not need to handle or care about node placement onto processes or inter-process communication. Neurons in NEST can be anything from simple point neuron models like the integrate-and-fire neuron to complex compartmental neurons, as long as they can be expressed as a single C++ class. Synapses can be either static or change their weight over time according to a plasticity algorithm. Examples of such algorithms are spike-timing-dependent plasticity (STDP), short-term plasticity (STP), or third-factor neuromodulated weight dynamics. Many different neuron and synapse models have been developed over time and are included in any distribution of the NEST source code.

NEST can be used from Python by means of a module called PyNEST that wraps the NEST simulation kernel, which itself is written in C++. Simulation scripts can then use functions like Create() and Connect() to create neurons and devices for stimulation and recording, and to connect these elements using different connection rules, respectively. A web-based graphical frontend called NEST Desktop simplifies the task of network creation by offering graphical metaphors and a point-and-click interface and has been especially useful in classroom scenarios. To keep the actual simulation of the neuronal network separate from the graphical frontend, NEST was extended by the NEST Server, which allows steering NEST via a RESTful API that listens on a specific TCP/IP port and maps incoming requests of the form http://localhost:5000/api/Createhttp://localhost:5000/api/Create to calls of the PyNEST API (the function Create() in the example).

When NEST is run in an MPI-distributed fashion, each process (or task, in MPI terminology) executes the same simulation script, but only creates its share of neurons, devices, and connections. The individual tasks also apply configuration changes only to local elements of the simulation and record data only from the entities they are responsible for. This is not a problem in many simulation experiments, where simulation scripts are run for the full simulation time and data is analyzed only after the simulation has finished and data from the different result files of the different processes has been manually combined. Due to the distributed nature of data collection in NEST, NEST Server originally only supported non-distributed simulation runs. To support the framework described in this work, NEST Server has now been extended to also support distributed scenarios by using a master-worker paradigm: The first MPI process (MPI rank 0, master) is responsible for both providing the RESTful API to clients, and forwarding all incoming commands to the workers (i.e., all MPI ranks but 0) and collecting their result data. In addition, the master process also participates in the neuronal simulation and thus also serves as a worker itself. A set of heuristics is used to combine and present the worker's response data to an outside caller as a consistent view that does not differ from one that the caller would see when only a single MPI process is used.

## 3. Software Architecture

The following provides the implementation details of a software architecture that integrates the Neurorobotics Platform and NEST Server for embodied simulations, supports browser-based online control of and interaction with experiments, and is highly scalable. This setup leverages a cloud computing infrastructure and HPC computing resources, both provided by EBRAINS. Despite the complexity of the architecture, automated deployment and online interactivity are provided through a dedicated graphical user interface available in the NRP frontend.

### 3.1. Infrastructure

**Figure 1:**
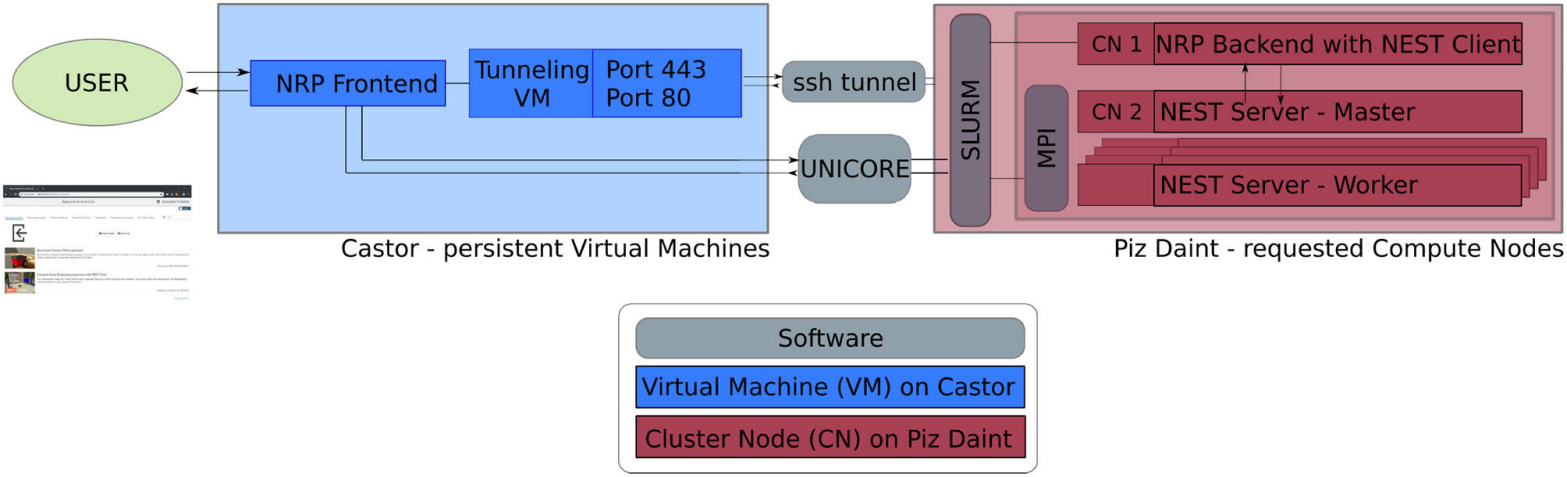
Software architecture. Persistent virtual machines are interfaced with requested compute resources in order to offer a flexible user interface with HPC resources. We use UNICORE as an interface to schedule compute jobs, and a SSH tunnel on demand establishes the bidirectional connection between NRP frontend and backend running on the two distinct computing systems.

The software service presented in this article is deployed across multiple compute systems at the Swiss National Supercomputing Center^3^ (CSCS). Therein, persistent virtual machines are used in order to let users interact with the NRP continuously and request HPC resources in the form of compute nodes. The overall architecture is illustrated in Figure 1. The NRP frontend and a proxy responsible for assigning NRP backends and handling REST calls are deployed on a virtual machine on the *Castor* cluster, while the actual embodied simulations (NRP backend and NEST brain simulation) are run on requested compute nodes of Piz Daint. For every NRP job, at least two compute nodes are requested, the first running the NRP backend, the second and any additional nodes running NEST Server. As such, the setup is fully scalable in terms of computing capabilities and is able to support custom large-scale embodied simulations.

The software interface between NRP frontend on Castor and NRP backend on Piz Daint compute nodes is instantiated on demand. We implement a UNICORE^4^ (Uniform Interface to Computing Resources) interface in the NRP proxy to interact with the SLURM workload manager (Yoo et al., 2003). UNICORE's REST API is used to request compute jobs, transmit configuration files and launch the NRP with NEST via startup scripts. We set up an SSH tunnel between the NRP frontend virtual machine and the cluster compute node running the NRP backend to enable bidirectional communication during runtime.

To facilitate fast and automated update cycles in a cloud infrastructure with our multi-component software architecture, we integrate all software components in Docker containers, in particular the NRP frontend, NRP backend and NEST Server. We use Jenkins with Ansible for Continuous Integration and Continuous Deployment (CI/CD); installation and Docker image instantiation on Castor is fully automated; new Piz Daint NRP backend images can be pushed to the Docker registry and then pulled to the Piz Daint login nodes using the Sarus container engine (Benedičič et al., 2019). The main advantage of this approach is the fast deployment of software improvements and new releases of the NRP and its components. This ensures forward compatibility of the platform during the ongoing NRP development. The architecture also allows multiple versions to be made available in the Docker registry so that custom software versions can be instantiated on demand.

### 3.2. Graphical User Interface

**Figure 2:**
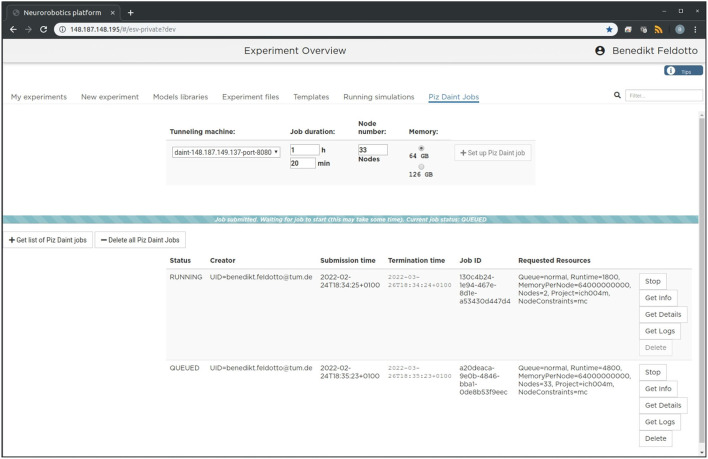
Graphical user interface. The large-scale simulation setup on HPC resources can be managed, accessed and controlled via a standard browser. We implemented a dedicated tab in the Neurorobotics Platform frontend that lets users parametrize supercomputing jobs and manage allocated resources. A new compute job running the NRP backend instance with distributed NEST can be requested and started with a single click from this frontend.

The setup is intended to enable future community access to an EBRAINS service that lets users experiment with large-scale embodied simulations without in-depth knowledge of the required underlying supercomputing infrastructure and architecture deployment. For this purpose, we implement a new section in the NRP frontend as shown in Figure 2, which can be accessed through a web browser. With it, users can request and launch the NRP on Piz Daint as compute jobs with customized resources, as well as manage instantiated jobs with running NRP instances. The job duration, compute node number and memory allocation can be customized depending on the duration and complexity of the experiments to be simulated. The frontend section also includes a list of past and running compute jobs, and lets users abort and inspect these during and after runtime. After starting the NRP backend distributed on requested Piz Daint compute nodes, it is accessible and can be selected just like any other backend running on virtual machines. Launching an experiment lets users interact with the rendered virtual environment and control the experiment interfaces and procedure runtime either graphically or programmatically via Python scripts in the NRP Virtual Coach. The Virtual Coach includes a Python REST interface to the NRP so that users can control simulations and observe its status via callback functions from a Python script. Additionally, experiment scripts can be modified programmatically and finally recorded data can be requested for postprocessing of experimental data.

### 3.3. NRP-NEST Coupling Architecture

**Figure 3:**
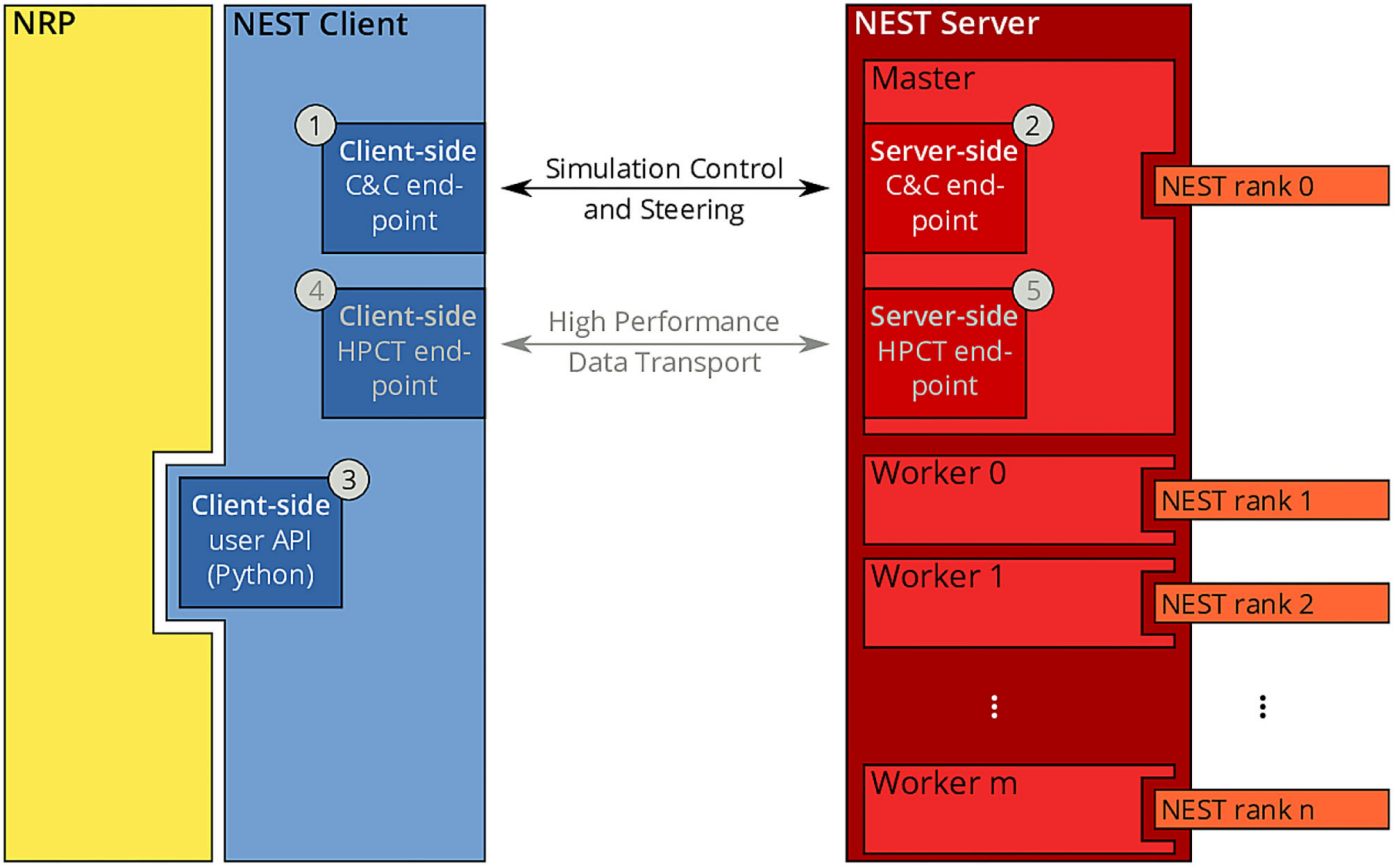
NRP-NEST client-server architecture. The NRP (yellow) connects to the NEST Client via its client-side user API for Python ③. The NEST Client (blue) provides a channel for talking to the NEST Server (between ① and ②) for simulation control and steering. A high-performance transport layer for data (between ④ and ⑤) is possible, but not yet implemented. The NEST Server (red) encompasses all NEST MPI processes (rank 0 to n), but only rank 0 (Master) offers the RESTful API visible to the outside world.

Since the beginning of the development of the NRP, NEST has been a first-class citizen in the NRP platform. It was initially integrated through a direct import of the Python module for NEST into the NRP CLE, which entailed a number of drawbacks in terms of code maintenance and distribution on multiple compute nodes of Piz Daint. To overcome the main drawbacks of the previous coupling, we started from the existing solutions and devised a new architecture based on the idea of separating NEST from the NRP by channeling all communication through the NEST Server and its RESTful API: instead of importing PyNEST directly, the NRP would only talk to NEST via HTTP requests and responses. The change to this new architecture constitutes a minimally invasive change to the NRP itself, as all code can be encapsulated in a new module that implements the NRP interface specification for integrating brain simulators on the one side, and a client for the NEST Server on the other. By having NEST run in its own process space, the issues related to code maintenance are eliminated, because NEST can run on any suitable Python version independently, and the version of NEST does not have to be taken into account by the NRP as long as the RESTful API of the NEST Server remains unchanged. The requirement for running distributed simulations of the brain simulation is naturally fulfilled in the new architecture as long as the MPI-enabled version of the NEST Server is used. The complete new coupling architecture is depicted in Figure 3.

Within the new client-server based architecture of the NRP-NEST coupling, the NEST Client Python module exposes a number of API functions in the client-side user API (③ in Figure 3) that allow to configure a neuronal network and the needed devices, run a network simulation for a given amount of time, and retrieve the recorded data. No actual computation takes place within the client. Rather, the latter forwards all operations to the NEST Server, which is based on a master/worker paradigm in which the *master* (MPI rank 0) provides a RESTful API to the NEST client and coordinates the workers by exchanging data and control commands with them. All MPI ranks (including rank 0) together execute the neuronal simulation. By virtue of this split in responsibilities, the actual details of the distribution remain completely transparent to the NEST Client.

In terms of deployment, the described separation between client and server allows execution of the components on different computing units. In particular, it is now possible to execute the NRP (including the NEST Client) and the NEST Server codes on different nodes of a given supercomputer or compute cluster, or even on completely independent machines. All NEST-related operations such as loading the network, stepping the simulation, creating devices and connectivity, are managed by the NEST Client that provides a set of user-friendly methods for all relevant operations.

The methods of the client-side API are (roughly speaking) just wrappers of the corresponding PyNEST functions. An example of such a method is get_kernel_status(), a call to which translates to an HTTP request for the URL host:port/api/GetKernelStatus, where *host* and *port* are the IP address of the machine on which NEST Server is running and the port it is listening on, respectively. Such a request results in a call of the PyNEST function GetKernelStatus() by the NEST Server. The return value of that function will be included in the response in the form of a JSON-encoded dictionary.

Below is a non-exhaustive list of the methods provided by the NEST Client client-side API to control and configure the simulation of the neuronal network in NEST:

- get_kernel_status(): access to NEST simulation parameters
- startup(): reset the kernel and set the number of threads and the simulation resolution
- load_network(): load a network in the form of a simulation script
- run_simulation(): drive a network simulation for a given amount of time
- create_device(): create a given number of network devices of a given type
- connect_device(): connect a device to a neuron using the provided connection parameters
- set_device_params(): set the given parameters on a device
- get_population_parameters(): retrieve parameters from a neuronal population

The second component of the coupling architecture, NEST Server, can be considered a language-independent interface to NEST that can be deployed either locally or on a remote machine as outlined above and in Section 2.4. Prior to any simulation, an instance of the NEST Server has to be started independently from the NRP and the NEST client with a degree of MPI parallelization that is suitable for the neuronal simulation at hand. As of writing, the NEST Server is fully integrated into the current release of NEST (Hahne et al., 2021) and can be either used after compiling the source from scratch, or from the NEST Docker image. All benchmark simulations presented in Section 6 have been realized using the containerized version of the NEST Server.

### 3.4. HPC Parallelization

Since the inception and widespread use of multi-socket/multi-core architectures several years ago, it has become more and more evident that a purely threaded application or one that purely relies on message passing for distributing the workload onto multiple processes is not sufficient for achieving optimal performance. Since then, the use of hybrid parallelization strategies that use threads within a CPU socket and message passing via MPI across CPU sockets and compute nodes has become the *de facto* standard for neuronal simulators. As this new paradigm has a high implementation complexity, many of the modern simulator codes shield the user from the details of the parallelization and provide suitable abstractions that also allow scientists not trained in computer science to use large-scale HPC machines efficiently.

For the NRP-NEST use case, we make use of the UNICORE REST API for requesting an individual number of compute nodes and running embodied simulations on compute resources customized to the user experiment. In contrast to a usual supercomputing job, in our case, not all compute nodes execute the same software but in fact run different sub-components of the overall architecture. After job approval, the execution of the architecture startup script is initiated via UNICORE, which launches the NRP backend, SSH tunneling service and NEST Server with workers on specific compute nodes. The general allocation layout is such that the user always requests *N* + 1 compute nodes, with the NRP and its NEST Client as well as the tunneling services started on the first node, and NEST Server on the remaining *N* nodes. This specific allocation of software components to compute nodes is done via the Slurm Workload Manager, assigning individual component execution scripts to the corresponding subsets of the overall allocated compute node list. Appropriate settings of thread pinning and process affinity are used to achieve good performance. For the deployment of NEST on Piz Daint for example, one MPI process is launched per physical CPU socket and set to use all of the 36 virtual and real cores by means of one OpenMP thread per core. NEST itself will then take care of the distribution of neurons and synapses onto the processes and threads by assigning neurons to threads in a round-robin fashion and allocating synapses on the process that is responsible for the post-synaptic neuron. We run two NEST workers on every individual compute node automatically (each assigned 36 CPU cores, see Section 5 for more details) to optimally use allocated compute resources. The described allocation scheme allows for fully customized scaling of computing capacity, which is only limited by the physical number of available nodes in the given HPC system.

## 4. Models and Setup

We implemented two different benchmark experiments in the Neurorobotics Platform to evaluate our software architecture: the first one is a rather synthetic balanced random network without any body connection; the second one is based on a biologically derived multi-region brain model connected to a virtual musculoskeletal rodent model.

### 4.1. HPC Benchmark With Balanced Networks

The random balanced network introduced by Brunel (2000) has been adopted by the NEST development community as a benchmark for large-scale simulations of spiking neural networks on HPC supercomputers (Morrison et al., 2007; Helias et al., 2012; Kunkel et al., 2014). This benchmark simulates a network with a large number of spiking neurons split into excitatory and inhibitory populations and random connectivity. The excitatory—excitatory synapses exhibit the multiplicative depression and power law potentiation model of Spike Timing Dependent Plasticity (STDP) described in the work of Morrison et al. (2007), while all connections targeting or originating from inhibitory neurons are static.

The number of neurons in the network corresponds to 11,250 multiplied by a scale parameter. The indegree of each neuron is fixed to 11,250 synapses regardless of the scale parameter. In this work, we use a scale factor of 20 yielding a network with 225,000 neurons and roughly 2.5 billion synapses. The network is simulated with a computational resolution of 0.1 ms for a duration of 1s. A four-wheeled Husky robot is loaded in a static virtual room but is left unconnected from the neural network and merely serves as a base workload for the NRP. In this benchmark setup, physics are simulated with Gazebo and the *ODE* engine.

### 4.2. Embodied Multi-Region Rodent Brain Experiment

The embodied multi-region rodent brain experiment aims to examine the dynamic mechanism of the cortico-basal ganglia-cerebellar-thalamic (CBCT) circuit in motor control through combined simulation of the brain model and the physical musculoskeletal model of a mouse. The embodied simulation includes 1,005,905 spiking neurons with 1,588,469,795 synapses in NEST. Neuronal output from the brain simulation controls the physical simulation of a mouse musculoskeletal model with 8 muscles in Gazebo and the *Simbody* physics engine. The NRP experiment view lets the user inspect, adapt and interact with the simulation online, **Figure 6** (bottom) shows the 3D rendering of the moving musculoskeletal body and the brain activity as a spike raster plot in the NRP frontend.

#### 4.2.1. The Multi-Region Rodent Brain Model

The CBCT model is based on the biologically constrained spiking network models of the cerebral cortex (Ctx), basal ganglia (BG), cerebellum (CB), and thalamus (TH) (Gutierrez et al., 2020). The numbers of neurons in the CBCT loop add up to more than 90% of the number of all neurons in rodents, primates, and humans (Azevedo et al., 2009; Herculano-Houzel, 2009).

The model consists of a reference cortical patch of 1×1mm^2^ and connected BG, CB and TH models with proportional number of neurons. In total, the model incorporates 1,005,905 neurons (Table 1). Simulations of such a large network combined with the musculoskeletal model requires efficient use of high-performance computing (HPC), especially for model optimization by repeated evaluations of the generated dynamics against experimental data. The NRP infrastructure provides the framework for managing access and execution by HPC. Moreover, it allows easy and efficient integration of the brain model with physical models with realistic behavioral constraints, which facilitates better validation and improves predictive power of the simulated models.

**Table 1:** Summary statistics of the 1×1 mm^2^ unit of the rodent brain model.

**Model**	**#Neurons**	**#Layers**	**#Neuron types**
M1 (Ctx)	58,805	5	19
S1 (Ctx)	94,396	7	22
VL (TH)	6,144	2	3
VM (TH)	6,144	2	3
BG	10,976	5	5
CB (M1)	414,720	6	6
CB (S1)	414,720	6	6
Total	1,005,905	33	65

*Parrot neurons and neurons instantiated by NRP devices as interface are not included*.

**Figure 4:**
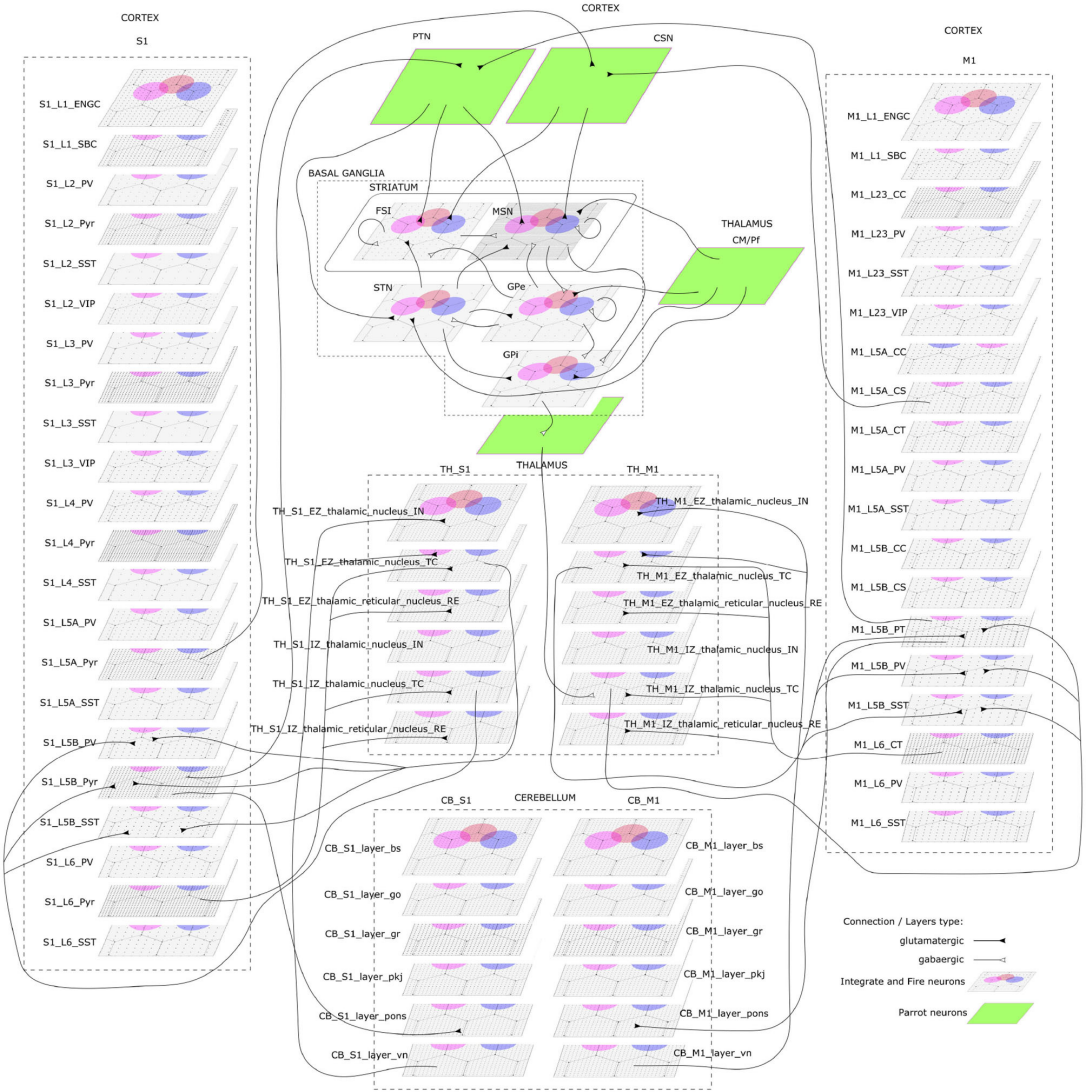
The cortico-basal ganglia-cerebellar-thalamic (CBCT) model of the rodent brain. The model includes the cortex (S1, M1), the basal ganglia (BG), the cerebellum (CB), and the thalamus (TH). Within each region, neural populations are topologically organized in 2D-layers of 1×1 mm^2^, with dots on their surface indicating the spatial allocation and density for each neuron type. Only main inter-regional connections are displayed for clarity. Layers in green correspond to the interface between different regions or simulated input, which are implemented using NEST's *parrot* neurons that just relay incoming spikes to multiple targets.

The CBCT model of the multi-region rodent brain (Figure 4) is composed of the following regional models:

*Cerebral Cortex*: The model incorporates the primary motor cortex (M1) and the primary somatosensory cortex (S1), based on previous works of Igarashi et al. (2019), Sun Zhe (2019), and Sun and Morteza Heidarinejad (2019). A unit model has the size of 1,000 x 1,000 x 1,400 (height x width x length) μm^3^ and contains six 2D sheets for the arrangement of neural populations in layers 1, 2/3, 4, 5A, 5B, and 6 based on reported cortical organization and experimental data (Lev and White, 1997; Weiler et al., 2008). Main neuron types are single bouquet (SBC) and elongated neurogliaform (ENGC) cells in layer 1; intratelencephalic (IT), parvalbumin-expressing (PV), and somatostatin-expressing (SST) neurons at layers 2/3, 5A, 6; and IT, pyramidal-tract (PT), PV and SST neurons in layer 5B (Jiang et al., 2013; Shepherd, 2013; Tremblay et al., 2016). The model also incorporates vasoactive intestinal peptide-expressing (VIP) neurons in layer 2/3 and connections based on Jiang et al. (2015). The S1 model Sun Zhe (2019) includes additional neurons in layer (L4).

For each layer (except layer 1), the numbers of excitatory and inhibitory neurons follow a ratio of 4:1, with a total number of about 58,000 and 94,000 neurons in 1×1mm^2^ for M1 and S1, respectively. The spatial organization is based on pseudo-randomly generated neuronal positions uniformly distributed within layer boundaries. Connections are generated using several 2D Gaussian probability functions describing distance-based connectivity between excitatory and inhibitory neurons, including recurrent connections, in different cortical layers. The relative magnitude of the connections, as well as the parameters of the Gaussian functions, are taken from reported laser-scanning photo-stimulation and patch-clamp experimental recordings (Song et al., 2005; Weiler et al., 2008; Lefort et al., 2009; Xu and Callaway, 2009; Kätzel et al., 2011; Apicella et al., 2012; Avermann et al., 2012; Jiang et al., 2013; Pfeffer et al., 2013; Xue et al., 2014; Lee et al., 2015; Pala and Petersen, 2015). Leaky-integrate-and-fire models with conductance-based synapses from the standard NEST model library are used. To achieve resting and functional states, neurons are stimulated by bias currents drawn from normal distributions with optimized mean and standard deviation parameters.

*Basal Ganglia*: The BG model is a topologically organized version (Gutierrez et al., unpublished) of previous works from Liénard and Girard (2014) and Girard et al. (2020). Fixed parameters were defined based on biological constraints, while free parameters were optimized against electrophysiological recordings. The total number of neurons sum up to around 10,000 for rodents following a reference 1×1mm^2^ cortical surface (Table 1), with most of them being medium spiny neurons (MSN). Neurons were spatially and uniformly organized in 2D space. Main inputs are from cortico-striatal neurons (CSN) and pyramidal tract neurons (PTN) in the cortex (M1 and S1) and the centromedian/parafascicular neurons (CMPf) in the thalamus (TH). The model considers glutamatergic excitatory inputs with AMPA and NMDA receptors and inhibitory inputs by GABA receptors. The model uses multi-synapse LIF neuron models from NEST. Connections follow the same architecture as in Girard et al. (2020), with specifications based on optimized bouton counts, and focused or diffused axonal domains. Simulation tests reproduced the firing rate of previous models in the resting state.

*Cerebellum*: The CB model consists of two regions connected with S1 and M1. Each cerebellar region is a corticonuclear microcomplex model developed in NEST based on the previous work of Yamaura et al. (2020). The cerebellum is modeled as seven stacked layers corresponding to 1×1mm^2^: upper and lower molecular layers, Purkinje cells, granular layer, deep cerebellar nucleus, and Pons (Eccles, 1967). The upper molecular layer was modeled as a group of four 2D layers of stellate cells, while the lower one as a single sheet of basket cells. Similarly, the granular layer was composed of eight sheets of granular cells and one sheet of Golgi cells. All other nuclei were modeled within single sheets. Number of neurons (Table 1) for each population were defined from previous data (Lange, 1975; Ito and Itō, 1984; Harvey and Napper, 1991; Heckroth, 1994). The cerebellum contains around 80% of the neurons (around 820,000 neurons) of our full brain model. Neurons were modeled as conductance-based leaky integrate-and-fire units, with parameters defined based on previous studies by Yamaura et al. (2020). Excitatory synapses were modeled as AMPA or NMDA, and inhibitory as GABA-A or GABA-B alpha-shaped synapses. Connections were settled according to known anatomical structures (Eccles, 1967; Apps and Garwicz, 2005; Barmack and Yakhnitsa, 2008), using 2D Gaussian functions for defining the spatial scope and connection probability between neurons. Most internal parameters such as capacitances, conductances, and synaptic weights were tuned and tested to reproduce electrophysiological and behavioral results on optokinetic responses, a cerebellum-dependent eye movement task based on the previous work by Yamaura et al. (2020). On the other hand, firing rates and synaptic weights for neurons in Pons were adjusted to obtain the mean firing rate of mossy fibers at 8 Hz, which resulted in reproducing plausible resting activity patterns. At that regime, granular cells revealed different temporal activity patterns, with random repetition of transitions between burst and silent states.

*Thalamus*: The TH model (Igarashi et al., unpublished) consists of two regions, ventral lateral nucleus and ventral medial nucleus, connected with M1 and S1, respectively. The individual thalamic nucleus is composed of excitatory and inhibitory zones receiving inputs from the cerebellum and basal ganglia. Each region-zone contains 1024 excitatory thalamocortical cells, 1024 inhibitory interneurons, and 1024 inhibitory thalamic reticular cells, arranged in a unit size corresponding to 1×1mm^2^ of the cerebral cortex. Thalamocortical cells and two types of inhibitory neurons are mutually connected, with no excitatory recurrent connections among thalamocortical cells.

*Inter-regional connections*: Inter-regional connections are set as topographic connections between two neural sheets. Major inter-regional pathways include: M1 L5A to BG Striatum, S1 L5A to BG Striatum, BG GPi/SNr to TH, M1 L5B to CB Pons, S1 L5B to CB Pons, CB deep cerebellar nucleus to TH, M1 L6 to TH, S1 L6 to TH, TH to L2/3 M1, and TH to L4 S1. A major challenge when integrating different models is to guarantee their optimized activities are maintained after combination. For instance, in the basal ganglia model, inputs from cortical models (M1 and S1, layers L5A and L5B) were adjusted to match the firing activity of PTN (pyramidal track neurons) and CSN (cortico-striatal neurons) inputs from Poisson spike trains used on the optimization of the isolated model. NEST's *parrot* neurons (models that just relay incoming spikes to their targets) were used to gradually replace Poison-based neurons by M1 and S1 based neurons. Thus, inputs involved in inter-regional connections were adjusted to those used on individual optimizations, using or not using parrot neurons on the connections.

#### 4.2.2. Resting-State Activity

**Figure 5:**
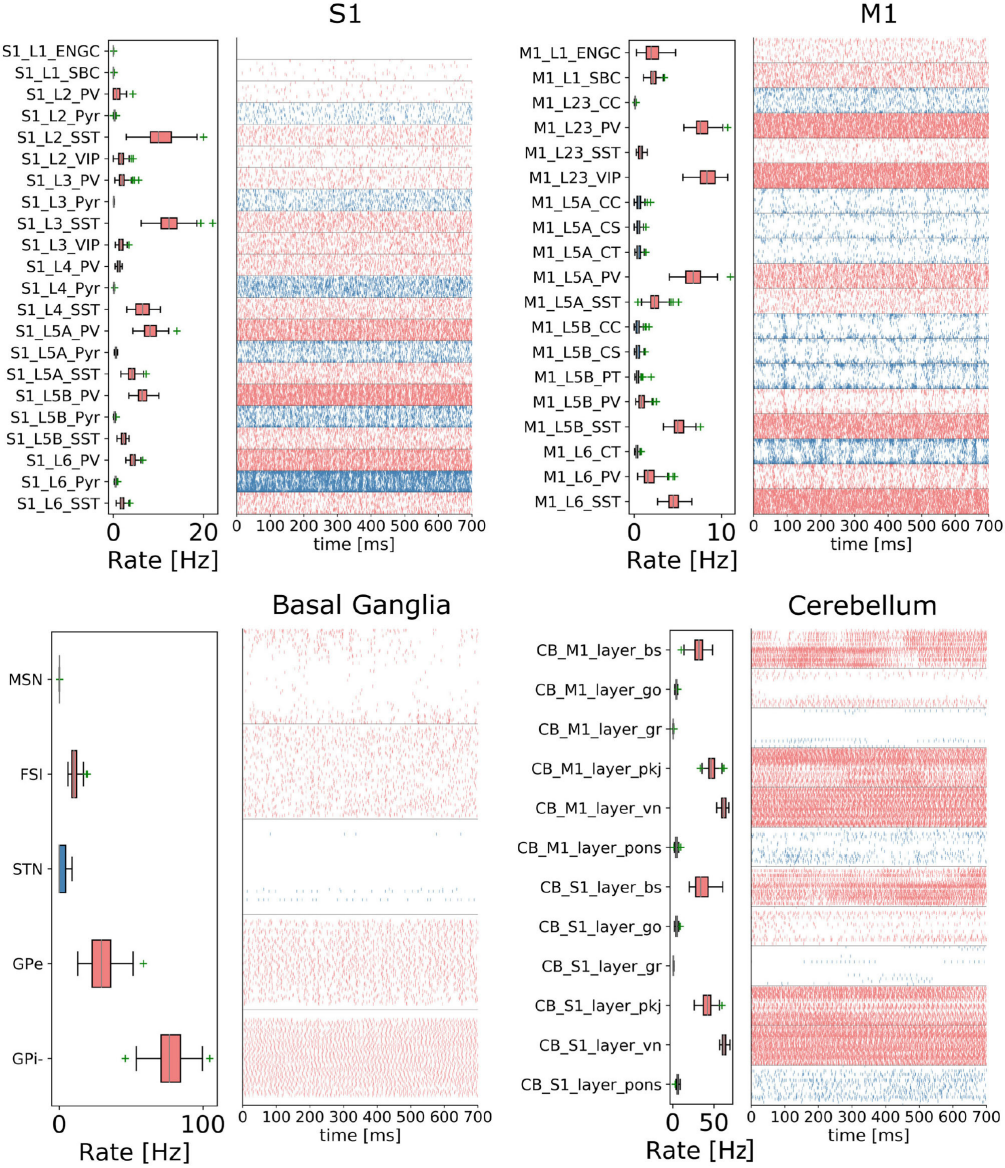
Resting-state of the CBCT circuit (Gutierrez et al., 2020). Spike rasters (right) and mean firing rate (left) per neuron type (thalamus activity is not displayed).

In order to reproduce resting-state neural activity that is simulated in this benchmark experiments, Poisson noise generators and constant current inputs were optimized to reproduce the average firing rates of individual neural populations based on physiological data (Figure 5). In S1, M1, and TH, neurons showed low-rate and irregular firing. Layers 5 and 6 in S1 generated gamma oscillation of around 40 Hz. Similarly, M1 bottom layers displayed oscillatory behavior. GPe and GPi/SNr in BG showed high-rate firing while others were kept low. In CB, Purkinje cells exhibited regular firing patterns, whereas granule cells emitted spikes sparsely. We acknowledge that the spiking activities of few neural populations could be slightly higher. While this model is a first version of the CBCT model used for benchmarking of the architecture presented here, a future release of our model aims to improve firing activities as well as other metrics.

#### 4.2.3. Embodied Simulation

The multi-region brain model is embodied into a simulated rodent musculoskeletal model and a virtual environment in the NRP. We replicate the physical experiment platform introduced in Mathis et al. (2017) as a simulation model *in silico* in the Neurorobotics Platform. In this model, the animal is held in place, rewarded by a Lickometer, and is able to manipulate a joystick to which additional forces can be applied via a linear solenoid magnet. We modeled a rodent housing, Lickometer and joystick in the Neurorobotics Platform using the Robot Designer^5^ plugin for Blender as part of the Neurorobotics Platform design tools. The joystick was connected to the world with two revolute joints representing two degrees of freedom. The mouse manipulated the joystick with its left forelimb, while a small effort of –0.001 Nm is applied to the joystick joint.

**Figure 6:**
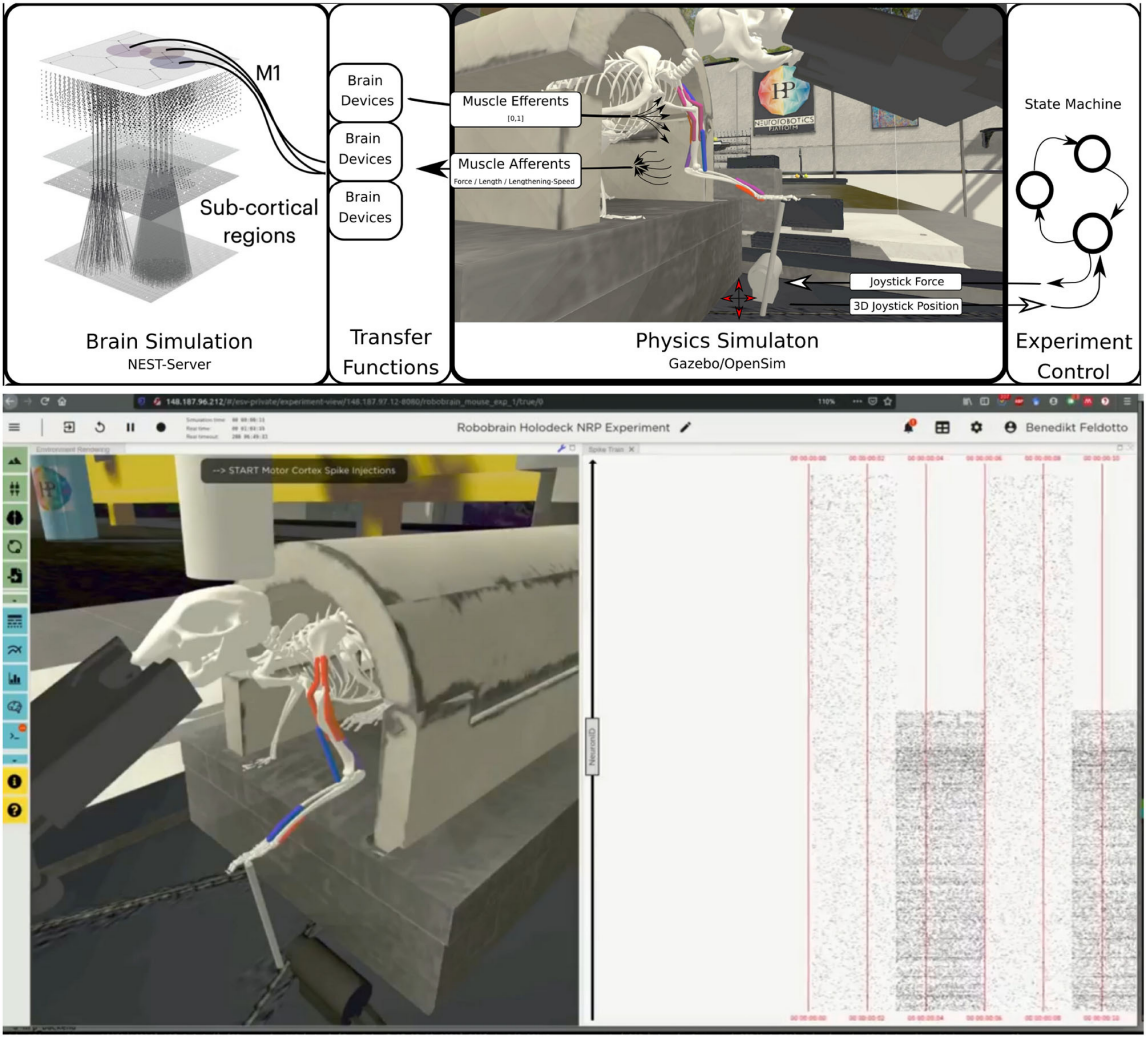
Embodiment of the multi-region rodent brain. (Top) The multi-region brain model is interconnected with the rodent musculoskeletal simulation via Transfer Functions. The readout rate of motor cortex populations actuates the rodent muscles moving the joystick forward. (Bottom) The user can interact with the simulation during runtime via the NRP frontend. Here we show the rendering of the simulated experiment on the left (muscle color coding: red–active, blue–not active) and spike trains of the motor cortex population on the right.

For the musculoskeletal system, we adapted a rodent simulation model that has been used in a stroke rehabilitation study in the Neurorobotics Platform recently (Vannucci et al., 2019; Allegra Mascaro et al., 2020). The skeleton thereof was modeled according to anatomical data and scans, and is an early version of the fully parameterized rodent model presented in Ramalingasetty et al. (2021). We anchored the rodent body model to the experimental apparatus, leaving only three moving segments of the left forelimb capable of movement: humerus, ulna/radius and the foot. Body and humerus were connected via two revolute joints, humerus and ulna/radius via one revolute joint and the foot was attached to the joystick via a ball joint with 3 degrees of freedom. With this configuration, the mouse was able to move the joystick in the forward/backward and lateral/medial directions. The skeleton was simulated as a rigid-body simulation with the *Simbody* multibody physics engine in Gazebo. We added 8 muscles to the forelimb joints, 2 for every rotation axis, with 2–5 muscle pathpoints each. Muscles were simulated with the OpenSim muscle implementation (Delp et al., 2007) and modeled with type “Millard2012EquilibriumMuscle” as described in Millard et al. (2013). Every muscle was actuated in normalized range [0,1]. Figure 6 illustrates the overall setup rendered in the Neurorobotics Platform frontend.

For the benchmark experiments in this study, we set up a naive representative brain-to-body connection. We connected one layer, the elongated neuroglia form cells of the motor cortex, to three muscles of the rodent model. For this we made use of *spike sinks* that read out the membrane potential of a leaky integrate-and-fire neuron with infinite threshold and connected to all neurons of the given population, and apply it as muscle activation signal. We also instantiated spike sinks for all layers in M1 and logged the corresponding voltages in the NRP frontend console for inspection. Additionally, we created spike sources consisting of Poisson neurons connected to all neurons of the given population for three layers of M1 including the elongated neurogliaform cells.

A base activation was sent to all muscles for the first 5 simulation steps (corresponding to 0.1s of simulation time) to stabilize the biomechanical model. Additionally, all spike sources including the source to the elongated neurogliaform cells of the motor cortex were set with a spike rate of 0 in every iteration. After 5 simulation steps, the clipped voltage readout of the elongated neurogliaform cells was applied as activation value to three muscles continuously (muscle activation in range [0,1]). Reaching 25 simulations steps (0.5s simulation time), we feed a rate of 5000.0 into the Poisson generators representing the spike source of the elongated neurogliaform cells. As a result, spike activity in this layer rose and the brain layer readout devices transmitted an increased muscle activation to the aforementioned three muscles. The overall experimental procedure resulted in a loose stabilization of the joystick in the first 25 simulation steps (0.5s of simulation time), followed by a forward motion of the rodent leg pushing the joystick forward as a consequence. Starting after 5 simulation steps a status message was shown in the frontend to indicate the current state of network input activation repeatedly to the user.

## 5. Benchmark Experiment Procedure

We ran the benchmark experiments on the XC40 multicore compute nodes of Piz Daint^6^. Each node of this partition is equipped with two Intel® Xeon® E5-2695 v4 18-core CPUs running at 2.10 GHz (2 x 18 cores, each having 2 virtual cores) and 120 GB of RAM. All experiments were executed with the Neurorobotics Platform version 3.2 and NEST version 3.0 (Hahne et al., 2021).

We executed one NEST process per CPU using all 36 (virtual) cores, hence at most two NEST processes per compute node. Every experiment ran for 1s of simulation time (assigned as a timeout to every experiment), consisting of 50 CLE step times of 20ms each (meaning that data between body and brain was exchanged every 20ms in simulation time). We carried out a series of benchmark experiments, starting with a single NEST process and scaling up to 64 processes, doubling the process number at each run. At the beginning of every benchmark series, we requested 33 compute nodes (1 NRP node, 32 NEST nodes) to ensure all runs in the same series that included a variable number of NEST processes were executed on the exact same node allocation. Every benchmark series was repeated multiple times with a new node allocation every time. Hereafter, we report the first 8 successful repetitions of every benchmark experiment. For reproducible experiment execution, we instantiated the NRP NEST setup with scripts directly on a Piz Daint login node. This experiment procedure allows us to access and collect all recorded performance data from different sources directly. After launching the framework, the NRP experiment was controlled from the main script via the NRP Virtual Coach. The experiment procedure is presented in the pseudocode below:

      **for** number of benchmark repetitions **do**
            Salloc - Request Piz Daint job with 33 nodes
            NRP - Launch backend container on compute node #1
            SSH - Set up SSH tunnel from NRP backend to frontend
            **for** number of NEST tasks n = 2^i^ **do**
                  NEST - Launch NEST with n processes on n/2 cluster nodes
                  Virtual Coach - Import benchmark experiment into NRP storage
                  Start experiment runtime timer
                  Virtual Coach - Launch NRP benchmark experiment
                  Virtual Coach - Start NRP benchmark experiment
                  **while** Experiment is running **do**
                        Virtual Coach - Wait for experiment to be finished
            **end while**
                  Stop experiment runtime timer
                  Virtual Coach - Save CLE profiler performance data
                  NEST - Save network performance data
                  Sacct - Save job performance data
                  Virtual Coach - Delete benchmark experiment from NRP storage
            **end for**
      **end for**

After every experiment we collected performance data from the NEST Server (neural network creation time, connection time and duration of last simulation step), the NRP CLE profiler (brain/robot/transfer function step times) and Slurm workload manager (memory and energy consumption). The total experiment runtime was tracked manually as shown in the pseudocode and the real-time factor was calculated as the quotient of CLE step simulation time to real time, whereas the CLE real time was taken to be the mean value of all CLE step times except the first one (indeed, the first CLE step executes initialization procedures, hence is significantly larger and does not reflect the CLE step time of the overall experiment). The code to run the benchmark and the results presented in this paper can be found in the GitHub repository https://github.com/HBPNeurorobotics/nestserver_benchmarks.

## 6. Results

We first executed and evaluated the HPC Benchmark experiment based on random networks without brain to body connection, and afterwards ran the multi-region rodent brain model with connection to the musculoskeletal rodent model. For a succeeding comparison between the benchmarks we added a third configuration that is a subset of the embodied multi-region rodent brain experiment with only the motor cortex as the brain model. This configuration shall not represent a biological simulation, but instead serves purely as a benchmark since a midsize brain in connection with the musculoskeletal model provides additional insights for the distribution of computation required for the simulation of brain and body.

Diagrams showcasing the compute node scaling use logarithmic scaling on the *x*-axis. We also present a linear expectation starting from the first point as the mean of all first data points without outliers that are not in range mean ±12%. The CLE profiler times represent a random benchmark repetition (here, the 4th), the *y*-axis is clipped as the initialization step takes significantly longer than usual runtime executions.

### 6.1. HPC Random Balanced Network Benchmark

**Figure 7:**
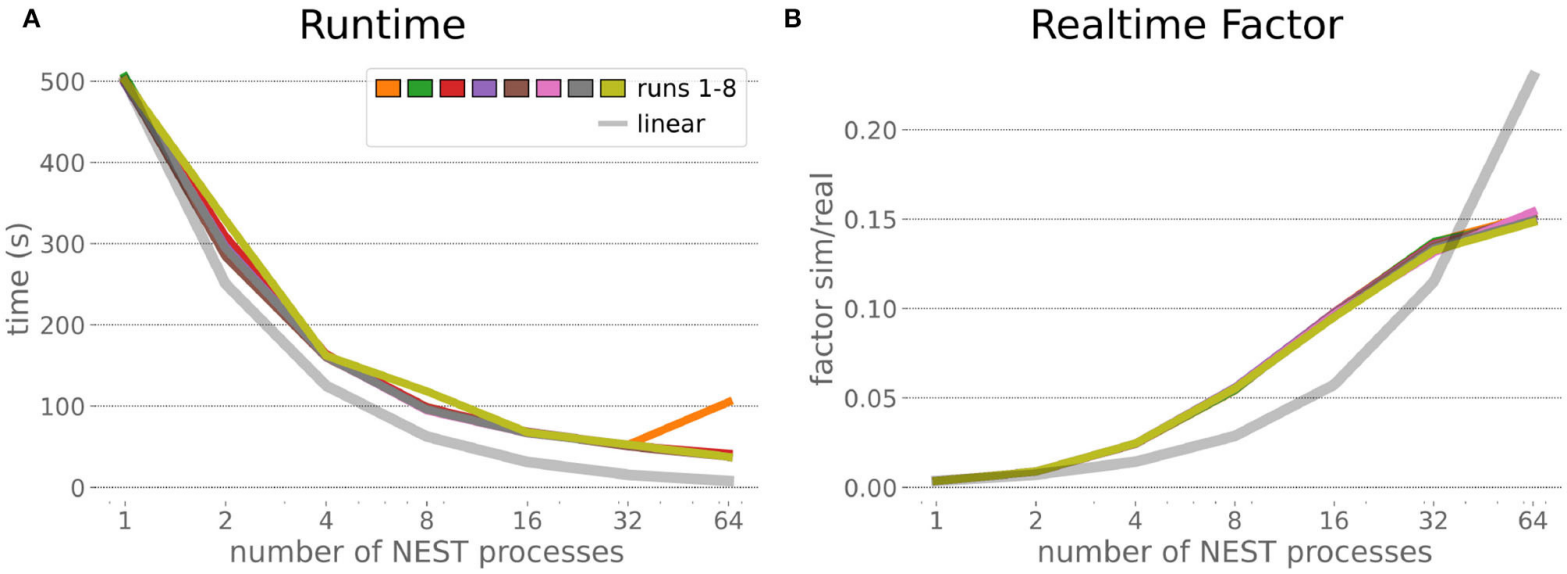
HPC Benchmark runtime and real-time factor. The runtime **(A)** can be reduced by a factor larger than 12 from 500s to about less than 40s by exploiting 64 NEST processes on 32 compute nodes compared to a single process. Simultaneously, the real-time factor **(B)** improves supra-linearly, but performance increases less significantly when using more than 32 NEST processes.

**Figure 8:**
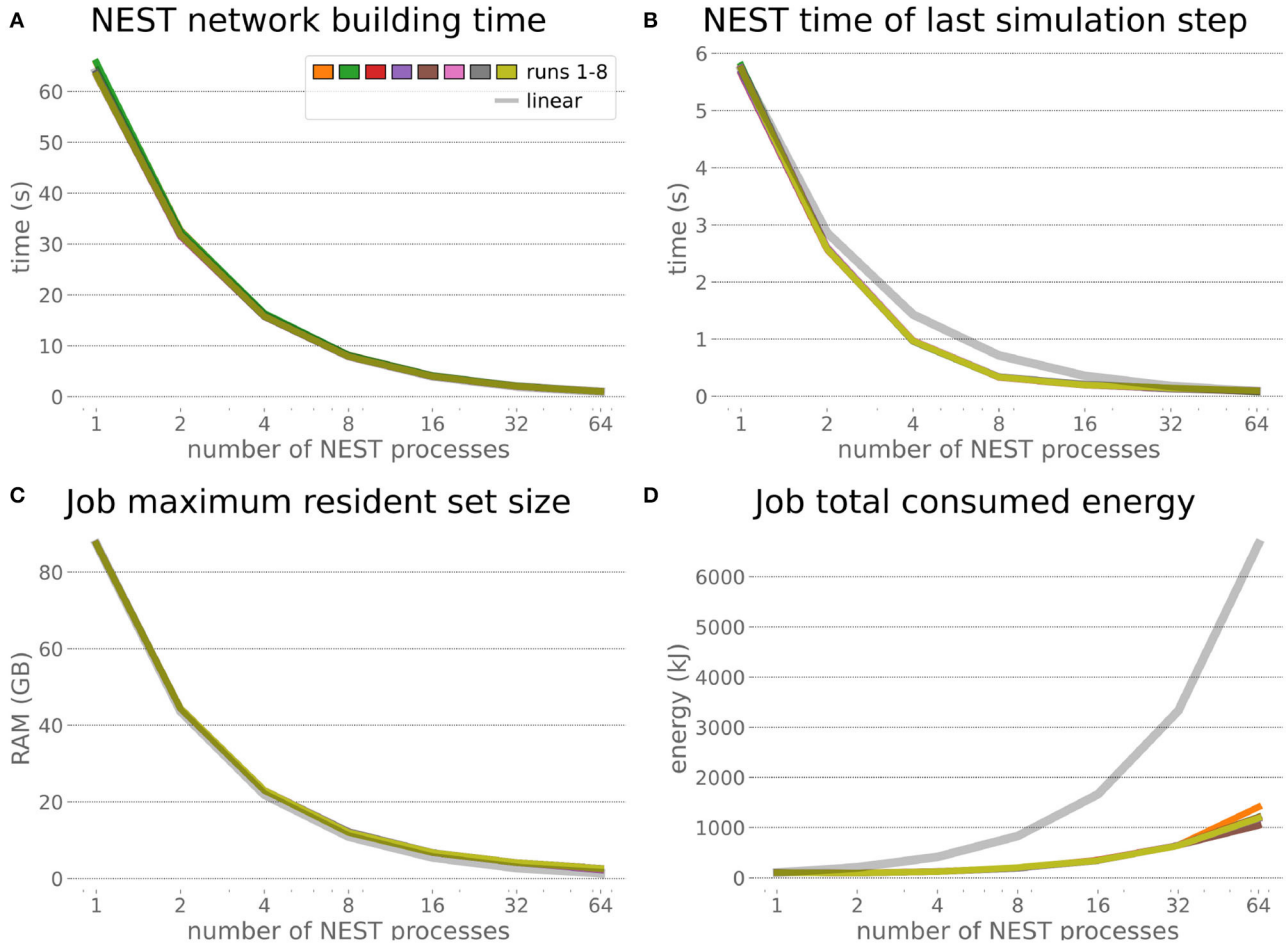
HPC Benchmark NEST times and workload manager characteristics. **(A)** Network building time (i.e., creating and interconnecting nodes) scales nearly linearly. The time to simulate the last brain step **(B)** scales even supra-linearly with 2–32 NEST processes. Similarly, the required memory per compute node **(C)** reduces close to linear, but the total energy consumed **(D)** by all tasks is only 12 times more for 64 NEST processes compared to 1 process.

**Figure 9:**
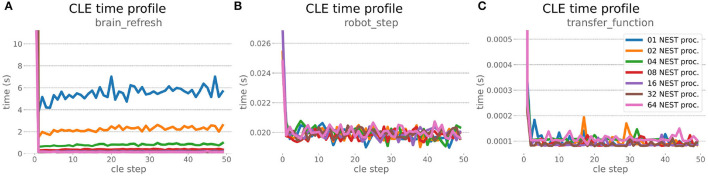
HPC Benchmark CLE profiler times. Robot **(B)** and transfer function **(C)** execution times do not change during the scaleup, as they are not parallelized and just run on the first compute node in the allocation. Both are neglectable compared to the brain step time **(A)** that runs faster with additional compute nodes. The first timestep includes additional initialization procedures and hence takes significantly longer than the usual runtime step time, in the diagrams we clip the y-axis for better visibility of the relevant runtime data.

The HPC Benchmark showed good repeatability with only a small variance between the benchmark runs. The runtime (Figure 7) could be reduced exponentially close to the linear expectation from about 500 to about 40 s, more than 12 times faster, when increasing the number of NEST processes from 1 to 64. The real-time factor increased exponentially first, but only up to about 8 processes; with more than 16 NEST processes a partial saturation appeared that resulted in an increase of the real-time factor up to 64 processes, albeit with a smaller slope. Overall the real-time factor could be increased from around 0.0036 to 0.150, a factor of more than 40. The NEST procedures scaled very well generally. The time required to build the network in NEST (i.e., creating and connecting nodes, Figure 8A) scaled nearly linearly. Simulation time (Figure 8B) scaled supra-linearly, but reached the same time performance as a linear scaling would have with 64 processes. Scaling up from 1 to 64 processes, the network building time could be reduced by a factor of about 61 and the time to simulate a brain step by about 60. The maximal memory required by a single HPC node (Figure 8C) could be reduced nearly linearly, from a maximum resident set size of about 87GB down to approximately 2.5GB. Along this scaling the amount of consumed energy (Figure 8D) did not increase linearly, but only by a factor of about 12 from 104kJ up to 1,200kJ. We observed that the three procedures of brain, robot and Transfer Function execution (Figure 9) all have an initial simulation step that takes significantly longer than the usual step time and hence is not considered in our analysis. In line with expectations, robot and Transfer Function execution step time varied over time but were not affected by the experiment parallelization. We observed a decrease of brain simulation time with increased numbers of NEST processes, with a slight overshoot in the first simulation steps and then stabilization at a mean value. As the robot and transfer function step times are relatively low in contrast to the brain execution, the latter one prominently defines the experiment runtime speed. Overall, the HPC Benchmark scales well, in most aspects nearly or supra-linearly, the one (beneficial) exception being the less-than-linear increase of consumed energy.

### 6.2. Embodied Multi-Region Rodent Brain

**Figure 10:**
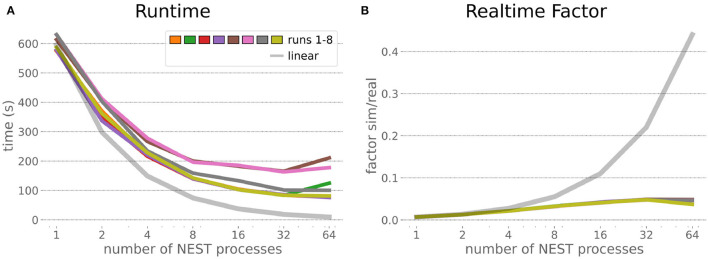
Embodied multi-region rodent brain benchmark runtime and realtime factor. Experiment runtime **(A)** shows a variability in repetitions, but can be improved exponentially by a factor of about 6 when scaling up to 64 NEST processes. The realtime factor **(B)** can be improved up to about 0.048, but starts saturating from 32 NEST processes onwards.

**Figure 11:**
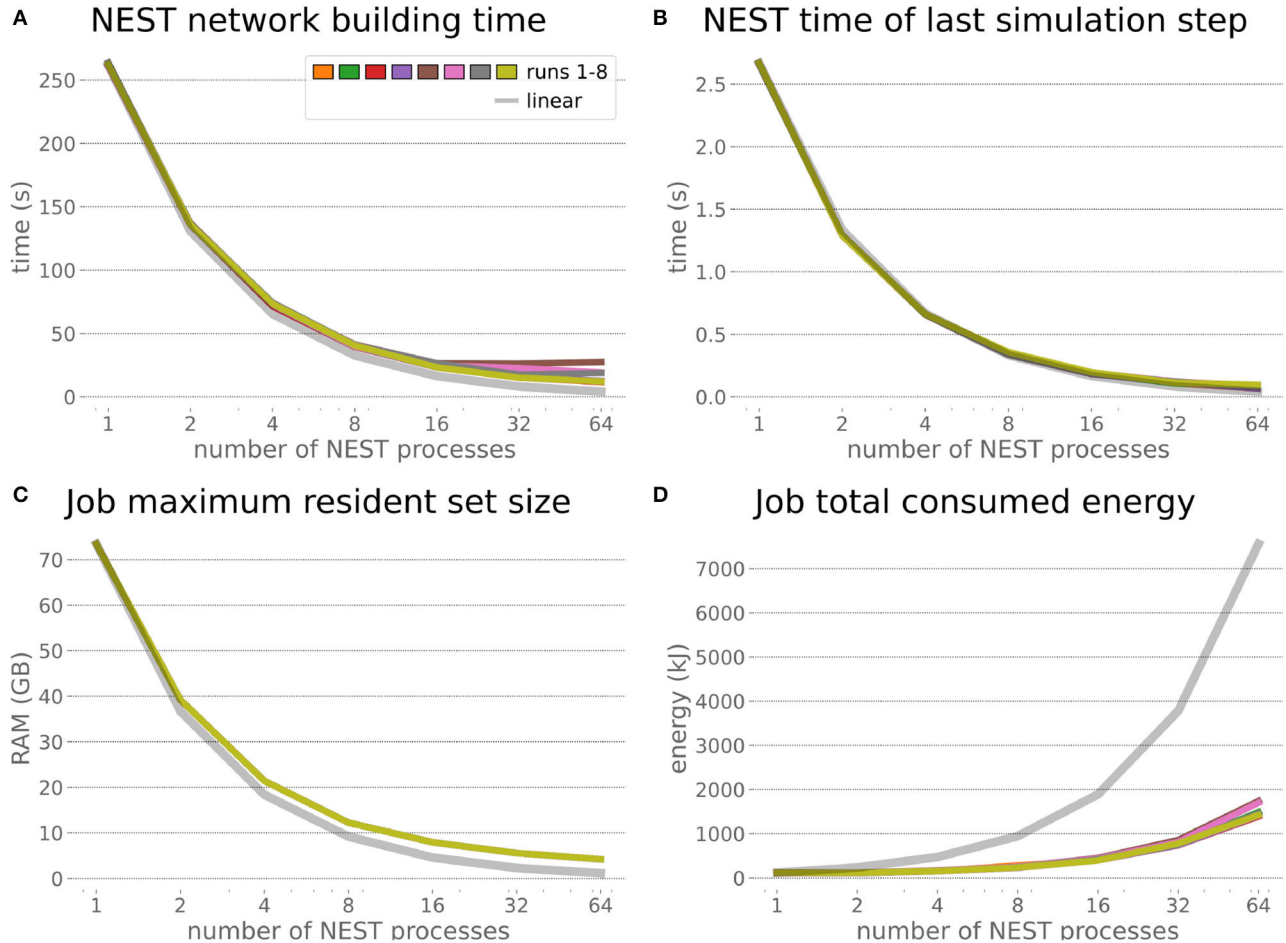
Embodied multi-region rodent brain benchmark NEST times and workload manager characteristics. NEST network building time **(A)** scales up close to linearly, NEST simulation time **(B)** nearly optimally linearly. Building time and simulation time shorten by factors of about 17 and 30, respectively. The required memory per node **(C)** for running the experiment scales close to linearly, but the total amount of consumed energy **(D)** only increases by a factor of about 12.

**Figure 12:**
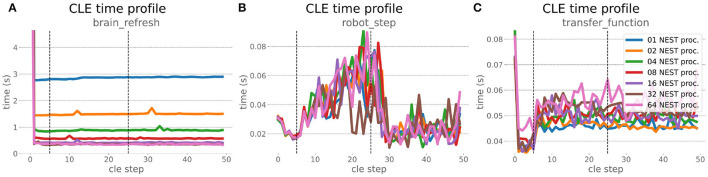
Embodied multi-region rodent brain benchmark CLE profiler times. Experiment execution has an impact on step times, setting the spike ratio of a motor cortex layer at step 5, and feeding the brain at 25 steps has a visible effect in Transfer Function **(C)** and robot **(B)** execution. Transfer Functions execute slightly slower with scaling up the experiment, but is still small compared to the large improvement in the brain execution times **(A)**. The first timestep includes additional initialization procedures and hence takes significantly longer than the usual runtime step time, in the diagrams we clip the y-axis for better visibility of the relevant runtime data.

The increased network size in the embodied multi-region rodent brain experiment compared to the balanced network benchmark (only 0.6 times the number of connections (1,588,456,283 vs. 2,531,475,000), but 4.8 times more network nodes compared to the balanced network benchmark (1,089,147 vs. 225,001) resulted in a larger overall experiment runtime as well as individual step execution times. The total runtime of the experiments showed a higher variance in repetitions (Figure 10) compared to the HPC Benchmark, which can be partly attributed to the larger execution times in general, and shows less than linear duration decrease but still a big improvement in time. The execution runtime could be reduced from about 600s down to about 100s, with two runs decreasing the runtime only down to about 200s during scale-up. Experiment runs that lasted longer usually took longer runtime in all node configurations in comparison to mean runtimes. The real-time factor of the experiment increased however only slightly and saturated using about 32 NEST processes, with a small decrease with 64 processes. This real-time factor was improved from about 0.0069 to 0.0480 (for 32 processes) during the scale-up, a factor of around 7. NEST procedures scaled exponentially (Figure 11), the simulation time (Figure 11B) close to linear, building time (Figure 11A) with a somewhat flatter decrease. The network building time could be sped up by a factor of more than 17 and the time for the last simulation step by a factor of about 30. The required memory did scale close to linear (factor of 17) to the number of nodes, and the consumed energy again increased far less than linearly, from about 120 kJ up to 1,450 kJ, by a factor of about 12 (Figures 11C,D). In contrast to the HPC Benchmark with balanced networks, execution times for robot and transfer functions changed over time (Figure 12), along with the scripted experiment procedure. We could clearly see an increase of computation time required by the Transfer Functions when a layer of the motor cortex was addressed with even a fixed spike rate of 0 after 5 execution steps. Changing the input rate to a higher value at 25 CLE steps did not have an impact on the execution time. We observed that the Transfer Function execution time increased slightly when scaling the experiment up to 64 NEST processes running on 32 different compute nodes. However, the execution time of Transfer Functions was still low compared to the brain execution time.

The robot execution time increased up to about 0.08s, reaching the highest values at around 25 simulation steps. It decreased afterwards at about 27 simulation steps and remained at a relatively low value of around 0.02s until the end of the benchmark time being 50 simulations steps. Brain step execution times showed less variability compared to the balanced network benchmark experiment, and were much higher in general than robot and Transfer Function execution times. Overall, the embodied multi-region rodent brain benchmark did not scale as well as the HPC Benchmark and showed more variability in terms of execution times. However, regardless of the network size, nearly all inspected timings still scaled close to linearly in relation to the number of nodes, which thus can be taken to speed up the experiment execution and decrease its runtime significantly.

### 6.3. Comparison

In order to optimize the experiments at scale, it is important to examine where the largest potential for improvements is, and what the costs related to scaling up execution will be. Therefore, we inspected the brain-to-robot compute time ratio as well as consumed node hours for all executed experiments and executed a third benchmark run that consisted of the embodied multi-region rodent brain setup, but with only the motor cortex as an active brain region.

**Figure 13:**
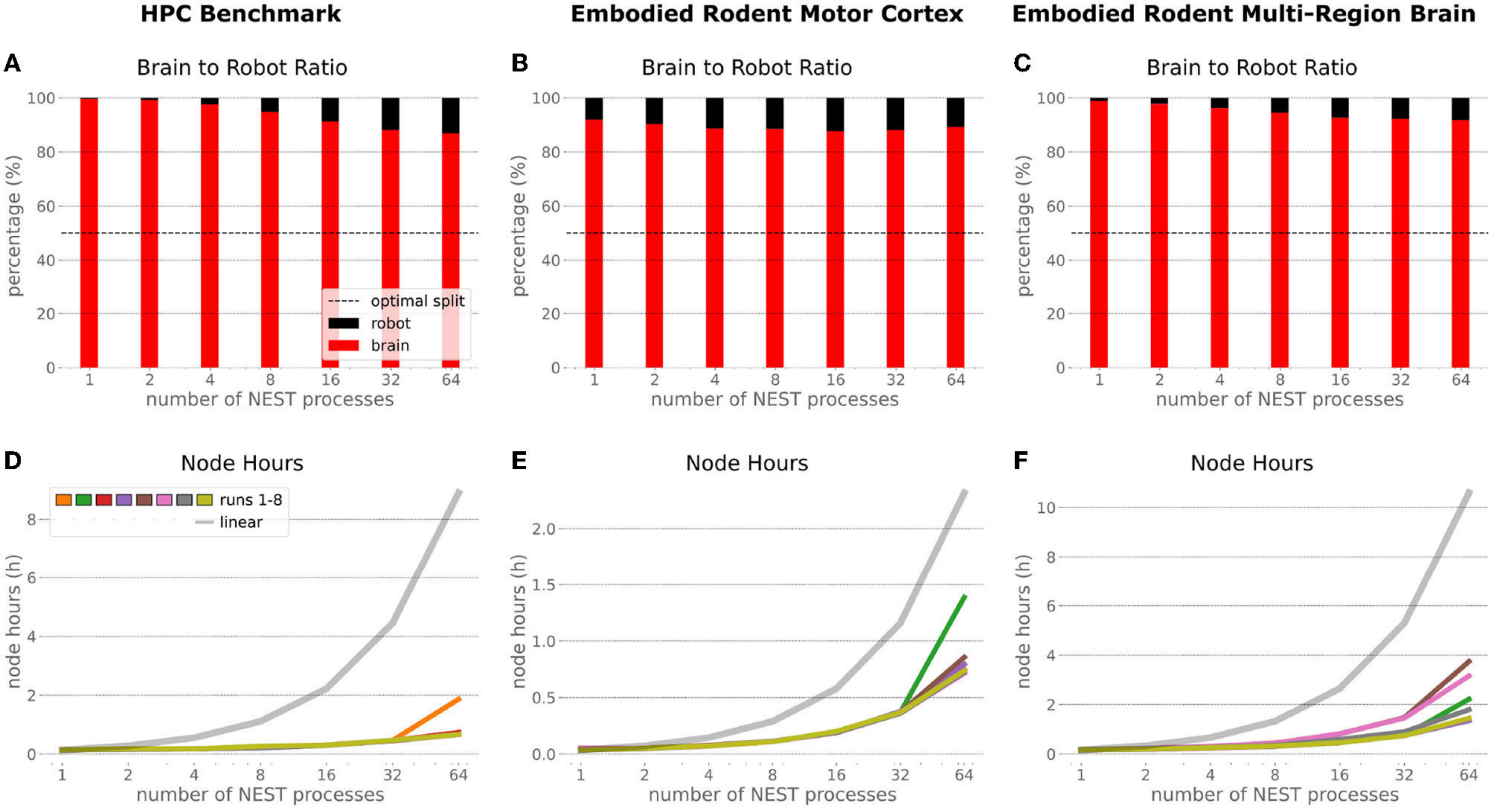
Comparison of benchmark experiments. Brain to Body step time ratio (mean values over all 8 benchmark repetitions) and node hours required to run the simulation for the HPC Benchmark, embodied rodent brain experiment with Motor cortex only and full embodied multi-region rodent brain experiment. For all experiments the brain execution takes longer than the robot simulation step **(A–C)**. This imbalance can be improved with our distributed architecture in particular for large neural networks as with the HPC Benchmark **(A)** and full embodied multi-region rodent brain experiment **(C)**. The required amount of node hours to run the experiments does not scale up linearly, it increases exponentially with small slope only. The embodied multi-region rodent brain experiment **(F)** requires the most node hours, but the required node hours increase by factors about less than 14, 35 and 23 for the HPC Benchmark **(D)**, embodied rodent brain experiment with only Motor cortex **(E)** and full embodied multi-region rodent brain experiment **(F)**, respectively.

In the NRP, at every CLE simulation step both robot and brain simulations are executed in parallel, and only after completion of these steps are Transfer Functions executed to process information to be communicated between both simulations. Obviously, when either one of the robot or brain simulation takes consistently longer to execute than its counterpart, that component becomes the target for optimizing the overall NRP simulation. In the top part of Figure 13, the ratio between brain and body simulation step time is visualized; times are mean values over all 8 benchmark repetitions. As both simulations are executed in parallel, the most efficient performance is achieved with both having the same execution time. For all experiments reported herein, the brain simulation step took longer than the robot simulation step. With our distributed architecture the ratio between the parallelized brain simulation step time and the (shorter) robot simulation step time improved as the brain simulation step time was reduced by distribution. This effect was less significant for small neural networks such as the embodied rodent brain experiment with a motor cortex only (B), but was very relevant for the full embodied multi-region rodent brain experiment (C) and balanced networks benchmark experiments (A). For both these large neural networks, the ratio between the two simulation time steps improved with the number of NEST processes, with the best result obtained for the 64 NEST processes we tested for these benchmark experiments. In the bottom part of Figure 13, the required node hours for every benchmark were calculated as the product of the pure experiment runtime, including experiment launch and execution but excluding the overall architecture setup and initialization, and the utilized number of nodes. As can be seen, the number of required node hours scaled less than linearly, i.e., exponentially but with small increments when scaling up the utilized node number. For the node hours of the HPC Benchmark (D) with balanced networks, the increase was by a factor of less than 14 from about 0.14 to 0.67/1.87 (best case/worst case), whereas for the embodied rodent brain experiment with Motor cortex only (E) it was by a factor of less than 35 from around 0.04 to 0.71/1.39, and for the full embodied multi-region rodent brain experiment (F) (which consumes the most resources), the consumption increased from 0.17 to 1.35/3.75 by a factor of less than 23. We also observed a higher variability of number of required node hours with increasing experiment complexity, and the embodied rodent brain experiment with only motor cortex showing the steepest increment.

## 7. Conclusion

In this paper, we presented a distributed architecture for large-scale embodied simulations of spiking neural networks, together with the results of benchmark experiments run on our setup. We sought to develop the software components of a future simulation service on the EBRAINS research infrastructure, while at the same time understanding the benefits and drawbacks of distributing simulations across nodes of the Piz Daint supercomputer.

For this purpose, we connected the Neurorobotics Platform for physics simulation via a REST interface to NEST for simulation of spiking neural networks used as brain models. We distributed this brain simulation across multiple HPC compute nodes via MPI parallelization, and thereby sped up both experiment loading and execution times. The proposed software architecture can be controlled via a browser-based graphical user interface integrated into the NRP frontend, and it extends across both persistent virtual machines and HPC compute nodes. To facilitate the technical implementation, we utilized standard tools such as Docker for containerization, Jenkins for automated deployment, and UNICORE for HPC job handling. This should enable easy transfer of the proposed architecture to other computing sites, in particular those that are part of the FENIX research infrastructure and/or EBRAINS. The presented setup is fully scalable, as the number of compute nodes involved in the simulation can be user-defined, and as multiple experiments executed on different job allocations can be launched simultaneously via the same front-end. Experiments run interactively, meaning that the user can join the simulations at any time via the web-based front-end, interact with the virtual agent and environment, or change the configuration of e.g., brain parameters, transfer functions and robot control.

We demonstrated the potential of our setup with two benchmark experiments scaled up from 2 to 33 compute nodes (1 to 64 NEST processes) using a balanced brain benchmark simulation and a multi-region embodied rodent brain model. We were able to speed up the total experiment execution time for the HPC Benchmark with balanced networks by a factor of up to 12, and for the RoboBrain experiment by a factor of about 6, thus demonstrating the potential benefits of distributing a brain simulation over multiple nodes, especially as it gets larger. Furthermore, the real-time factor could be improved, particularly for the benchmark based on balanced networks. It saturated with more than 32 nodes, however, potentially indicating that scaling-up is not always beneficial in cases where the overhead required for communication with all compute nodes at every simulation step becomes significant in relation to the compute load on each individual node. Nevertheless, the improvements we could demonstrate with distribution in terms of real-time factor lay the foundation for large-scale experiments that could otherwise not be carried out interactively due to their slow execution.

With both benchmark experiments we also demonstrated that NEST scales linearly, or near-linearly when parallelizing across 1–64 processes in terms of network building and simulation time. Regarding the cost of distribution for the benchmark experiments, we found that both energy consumed and compute node hours required scale sub-linearly and hence provide a strong argument for distributed simulations. The parallelization of the brain simulation accounts for better usage of computation time in our examples, as both brain and robot simulation are executed in parallel at every simulation step in the NRP, and thus can be better aligned with each other since the brain simulation is consistently the limiting factor. This ratio may even improve for a more complex rodent model physics simulation with more muscle actuators.

When NEST is run in a stand-alone fashion, it shows excellent scaling (Kunkel et al., 2014; Jordan et al., 2018) and is even able to achieve sub-realtime performance for certain models (Kurth et al., 2022). There are several reasons why the scaling is not at this level for the use-case presented in this article. First, due to the synchronization between network and physics simulation, NEST is executed in steps of 20 ms in the NRP and such stepped simulations are inherently more expensive due to the increased function call overhead and the fact that data-structures have to be paged in and out much more frequently rather than operating on them in a more continuous way. Second, both simulators are executed in parallel, but the data exchange still needs to be executed sequentially, which adds to the raw neural network simulation times. Third, we chose a REST-based communication interface between the NRP and NEST Server for the first version of the interface presented here, since it is functionally complete and has successfully been used in other contexts. This communication via text-based data representations (JSON over HTTP) is obviously inefficient compared to lower level protocols such as Google's Protocol Buffers^7^ or Cap'n Proto^8^. We are aware of this restrictions and already working on moving to more optimized communication methods with higher bandwidth and lower latency (e.g., Insite framework). It is worth noting here, that the current setup will support any future NEST improvements transparently, as long as these do not change the NEST Server API.

Overall, we approached saturation when scaling up to about 64 NEST processes. For the larger embodied multi-region rodent brain experiment, this saturation was visible with 32 processes in terms of both runtime and real-time factors. With both benchmark experiments we demonstrated that a scale-up to about 8 nodes could bring a significant performance improvement in terms of initialization and runtime of experiments, at the cost of only few additional node hours and concomitant energy consumption. With more compute nodes, additional improvements were possible, albeit less significantly and at a slightly higher cost. We proved the repeatability of our results by executing every benchmark experiment 8 times. Even though we ran our benchmark experiments with only 1s simulation time in order to save energy, we think it is safe to assume that our results will scale, as we showed relatively stable simulation execution step sizes in the CLE profiler data.

The setup we presented here is intrinsically highly scalable, insofar as the number of compute nodes can be passed as a parameter and can be much larger than the 33 compute nodes used for the presented benchmark experiments. While we simulated a multi-region brain model consisting of about one million neurons in these benchmark experiments, a biological mouse brain is assumed to have around 70 million neurons, and therefore another scale-up by a factor of 70 would be needed to simulate such a brain at the naturalistic scale. We are currently not aware of any embodied brain simulation model with larger scale that is implemented with the given software tools and that we could have used for our benchmarks, but such models are clearly part of future work. While the benchmarks presented saturate in terms of performance with about 32 or 64 compute nodes, it has been demonstrated that NEST scales well above that with a larger number of CPU cores (Kunkel et al., 2014; Kurth et al., 2022). Knowing that there are 1813 available multicore compute nodes on the Piz Daint supercomputer, we could approach this simulation scale with our current setup with just a parameter change—and a good budget. The Piz Daint supercomputer also provides GPU compute nodes that are well known for efficient parallel computing. However, Kurth et al. (2022) show that NEST distributed on CPU cores is faster and more energy efficient than any neuromorphic and GPU based simulation known to us.

A wide variety of experiments are supported with our setup, as it easily enables one to add additional muscles for the rodent model (e.g., a freely running mouse with additional muscles), use a different musculoskeletal model altogether (Human, monkey) or use NEST-based spiking neural networks to control a robotic system. In particular, we posit that integration of a detailed model of spinal cord circuitry with the whole-brain model presented herein would be highly relevant in order to investigate *in silico* experiments related to motor control, neurotechnology and neurorehabilitation. The proposed setup is therefore extremely versatile and can support research efforts in multiple high-impact fields, such as neuroscience, robotics and neuromorphic computing.

More generally, the present work lays the foundation to address the scientific dimension of large-scale brain simulation in addition to its technical one. The scientific investigation and validation of the dynamics emerging from the interaction of several types of neurons is indeed critical, as well as the optimizations of the high-degree-of-freedom parameter space of network models. Biological constraints were incorporated in the different regions of the CBCT model; however, once interconnected, the model as a whole requires a proper framework for systematic simulation with additional naturalistic constraints or boundary conditions, i.e., a body, for relevant experimentation on cognitive and motor functions. The reference model size defined herein in relation to the 1×1mm^2^ cortical patch provides an initial setup for starting such validation process. However, the ultimate goal is the simulation of the full-brain network. Previously, large-scale simulations of the CBCT model were performed on the decommissioned K computer (Miyazaki et al., 2012) using NEST 2, reaching a network size of 7 × 7mm^2^; thus, 51 million neurons, more than a single hemisphere of the mouse brain (Gutierrez et al., 2020). The new NEST 3 (de Schepper et al., 2022), NRP, EBRAINS HPC infrastructure, as well as the Fugaku supercomputer (Sato et al., 2020), provide a promising new horizon for 1:1 scale simulations.

In summary, we introduced a versatile NRP-based setup that supports embodied large-scale brain simulations. It can accommodate spiking neural networks implemented in NEST and connected to customizable musculoskeletal systems or robotic agents. We tested it with several models of spiking neural networks, including a highly complex multi-area brain model, thus demonstrating the capacity of this setup for *in silico* closed-loop neuroscience at scale. Importantly, it leverages the HPC capabilities of a supercomputer while supporting online interactivity with the ongoing simulations. With this setup, we thus lay the foundations toward the democratization of *in silico* behavioral experiments with large-scale multi-area brain models. Indeed, the *raison d'être* of this work is to remove some of the main entry barriers that prevent computational neuroscientists or neuromorphic engineers from testing the functional capabilities of their models through embodied simulations, and make it as easy as possible for them to leverage HPC infrastructures without being a power user thereof. In order to achieve this vision, the upcoming development efforts will focus on integrating the setup fully into the EBRAINS research infrastructure, especially in terms of federated user resource management and the creation of a dedicated service account. With this, it is our hope that this work will not be yet another attempt at simulating the brain, but a blueprint that can be reused by many, and an enabling technology for the concept of embodiment to gain traction in the neuroscience community.

## Data Availability Statement

The datasets presented in this study can be found in online repositories. The names of the repository/repositories and accession number(s) can be found below: https://github.com/HBPNeurorobotics/nestserver_benchmarks.

## Author Contributions

BF and FM conceptualized the benchmark study and orchestrated the implementation. BF, CB, UA, ER, VV, VZ, AK, and FM implemented necessary features in the Neurorobotics Platform. CB, AU, FC, and CM supported the implementation and execution on HPC nodes of Piz Daint and virtual machines on Castor. JE, CJ-R, WK, and AM implemented the parallelization of NEST on multiple cluster nodes. JE and CB implemented the client-server interface between the NRP and NEST. CG, ZS, HY, MH, JI, TY, and KD implemented the multi-region rodent brain model. BF integrated the multi-region rodent brain model into the NRP, adapted rodent and environment model and implemented the brain to body interconnection. BF implemented the NRP frontend GUI to launch the NRP on Piz Daint compute nodes. BF, JE, and CJ-R executed the benchmark experiments. Everyone contributed to analyzing the benchmark data and writing the paper. All authors contributed to the article and approved the submitted version.

## Funding

This work received funding from the European Union's Horizon 2020 Framework Programme for Research and Innovation under the Specific Grant Agreements No. 785907 (Human Brain Project SGA2) and no. 945539 (Human Brain Project SGA3). We acknowledge the use of Fenix Infrastructure Resources, which are partially funded from the European Union's Horizon 2020 research and innovation programme through the ICEI project under the grant agreement no. 800858. This project was supported by MEXT as Program for Promoting Researches on the Supercomputer Fugaku (hp200139, hp210169). Part of this study was supported by MEXT KAKENHI grant no. 17H06310.

## Conflict of Interest

The authors declare that the research was conducted in the absence of any commercial or financial relationships that could be construed as a potential conflict of interest.

## Publisher's Note

All claims expressed in this article are solely those of the authors and do not necessarily represent those of their affiliated organizations, or those of the publisher, the editors and the reviewers. Any product that may be evaluated in this article, or claim that may be made by its manufacturer, is not guaranteed or endorsed by the publisher.

## Footnotes

^1^ https://fenix-ri.eu/

^2^ https://www.nsgportal.org/

^3^ https://www.cscs.ch/

^4^ https://sourceforge.net/projects/unicore/

^5^ https://github.com/HBPNeurorobotics/BlenderRobotDesigner

^6^ https://www.cscs.ch/computers/piz-daint/

^7^ https://developers.google.com/protocol-buffers

^8^ https://capnproto.org/
